# Draft Genome Sequence of the Hydrogenogenic Carboxydotroph Moorella stamsii DSM 26271

**DOI:** 10.1128/genomeA.00345-18

**Published:** 2018-05-03

**Authors:** Anja Poehlein, Tim Böer, Kerrin Steensen, Rolf Daniel

**Affiliations:** a Genomic and Applied Microbiology and Göttingen Genomics Laboratory, Institute of Microbiology and Genetics, Georg-August University of Göttingen, Göttingen, Germany; b Members of the Applied Bioinformatics in Microbiology Course of the Microbiology and Biochemistry MSc/PhD Program, Georg-August University of Göttingen, Göttingen, Germany

## Abstract

The spore-forming, thermophilic, and obligate anaerobic bacterium Moorella stamsii was isolated from digester sludge. Apart from its ability to use carbon monoxide for growth, M. stamsii harbors several enzymes for the use of different sugars. The draft genome has a size of 3,329 Mb and contains 3,306 predicted protein-encoding genes.

## GENOME ANNOUNCEMENT

Moorella stamsii DSM 26271 is an obligate anaerobic, thermophilic, and endospore-forming bacterium belonging to the class Clostridia (1). Its ability to use carbon monoxide is coupled to equimolar formation of hydrogen and carbon dioxide (2). M. stamsii is reported to occur in single or paired straight rods. Gram stain results of this species vary, although Gram stain results for other Moorella strains are positive (2). Analysis of the 16S rRNA gene revealed that M. glycerini is the closest related organism of M. stamsii. The isolated strain DSM 26271 was derived from sludge of a municipal solid waste digester in Barcelona, Spain (2).

Extraction of chromosomal DNA of M. stamsii was performed using the MasterPure complete DNA purification kit according to the instructions of the manufacturer (Epicentre, Madison, WI, USA). Isolated DNA was used to generate paired-end sequencing libraries and subsequently sequenced using the MiSeq reagent kit version 3 and the MiSeq sequencing platform, as described by the manufacturer (Illumina, San Diego, CA, USA). Obtained reads were quality filtered by using Trimmomatic version 0.36 (3), which resulted in 1,725,652 paired-end reads. The SPAdes assembler version 3.10.0 (4) was used. Assembly yielded 82 contigs (>500 bp) with an average 107-fold coverage. The size of the draft genome was 3,329 Mb with a GC content of 53.81%. FastQC version 0.11.5 was used to investigate the quality of the sequence, and QualiMap version 2.1 (5) was employed to validate the assembly. Finally, the genome was annotated using the Prokka tool (6).

The Prokka annotation of the M. stamsii DSM 26271 genome resulted in 2,515 protein-coding genes with a predicted function and 791 hypothetical proteins. Furthermore, the draft genome contains 49 tRNAs, 3 rRNAs, and several antibiotic resistance genes. M. stamsii DSM 26271 can utilize various sugars, such as mannose, xylose, and galactose. Correspondingly, several genes associated with sugar metabolism, such as genes encoding a putative mannose-6-phosphate-isomerase, a xylose isomerase, and a galactose-1-phosphate uridylyltransferase, were predicted in the genome. In addition, M. stamsii DSM 26271 is able to use carbon monoxide as a sole carbon source by employing a water-gas shift producing carbon dioxide and hydrogen (2). Accordingly, a carbon monoxide dehydrogenase/acetyl-coenzyme A (CoA) synthetase gene cluster can be found as described for other autotrophic acetogens, such as Clostridium aceticum (7). The acetyl-CoA synthetase gene cluster shows the same arrangement as the Wood-Ljungdahl gene cluster found in M. thermoacetica DSM 521^T^ (8) and M. mulderi DSM 14980^T^ (9).

### Accession number(s).

The whole-genome shotgun project reported here has been deposited at DDBJ/ENA/GenBank under the accession number PVXL00000000. The version described here is the first version, PVXL01000000.
